# 

**Published:** 1892-05

**Authors:** 


### CONTENTS.

Original Articles—Wounds and Diseases Involving Both Abdomen and Thorax—By J. McFadden Gaston, M. D., Atlanta, Ga............261
Antiseptics in Railroad Surgery—By C. H. Richardson, M. D., Montezuma, Ga............268
A Cold—By J. J. Waller, M. D., Oliver Springs, Tenn............272
Correspondence—New York Letter............274
Society Notes—Clinical Society of Maryland............278
Georgia State Medical Association............285
Book Reviews—The Principles and Practice of Medicine, Osler............297
Medical and Surgical Gynæcology, Pozzi............297
Surgery. Its Theory and Practice, Walsham............298
Selections and Abstracts—Early Expulsion of the Placenta............300
Amenorrhœa of School Girls............300
The Spread of Syphilis by Cigars............301
Editorial—The Georgia Medical Association............302
The Doctor vs. The Minister............304
The Central R. R. Surgeons' Association of Georgia............306
American Academy of Medicine............307
American Medical Association............308
Prescription Department............312
Special Notes............310

